# Patient-reported outcome and prognostic factors for survival in patients with metastases in soft tissue treated with palliative radiotherapy

**DOI:** 10.1016/j.tipsro.2026.100406

**Published:** 2026-04-19

**Authors:** Lijanne Otto-Vollaard, Marcella Cramer, Erik van Werkhoven, Ingrid Lokken, Ilse M.N. de Pree, Joost J. Nuyttens

**Affiliations:** Department of radiotherapy, Erasmus MC Cancer Institute, Rotterdam, the Netherlands

**Keywords:** Soft tissue metastases, Palliative radiotherapy, Prognostic factors, Outcome, Survival

## Abstract

**Purpose:**

To determine the overall result of treatment with palliative radiotherapy for patients with soft tissue metastases and to identify prognostic factors for overall survival.

**Methods and materials:**

From September 2015 until April 2023, 120 patients were included. Treatment outcome was reported via telephone consultations at four and eight weeks after the last fraction. Patients who reported benefit were classified as responders, while those without benefit were classified as non-responders.

**Results:**

The most frequent complaints were pain (n = 104), swelling (n = 20) or functional disability (n = 19). There was a decrease or disappearance in 61% of complaints 8 weeks after last fraction of treatment. Median overall survival was 4.3 months (95% CI 2.4–9.3). 26 patients (22%, 95% CI 14%–29%) died within 2 months. From the 94 patients alive two months after treatment 66 responders had a significant better survival compared to 25 non-responders (6.6 vs 3.8 months, p = 0.005).

Multivariable analysis identified 5 prognostic factors for overall survival: PTV-volume, time from diagnosis to treatment, total number of metastases, systemic treatment options after radiotherapy, and number of organs.

**Conclusion:**

Palliative radiotherapy for patients with soft tissue metastases provided benefit in 61% of complaints that were the reason for treatment. Responders had a significantly better survival than non-responders. Although 5 prognostic factors for survival were identified, further research is needed to identify patients who benefit from palliative radiotherapy.

## Highlights

• Pain disappeared or decreased in 61% of patients.
• Responders had a median overall survival of 6.6 months compared to 3.8 months for non-responders.
• Five significant prognostic factors for survival were found.

## Introduction

Survival of patients with cancer has been increasing worldwide [1], due to developments in treatment, including immunotherapy, new surgical techniques and multimodality therapy [2], [3], [4]. However, these developments have also led to an increase in the number of patients with metastases [5], [6]. Unlike primary tumors, which can often be cured with local therapies such as surgery and radiation, metastatic cancer is a systemic disease that affects organ function and remains the most important cause of cancer-related death [7]. Metastases commonly affect lungs, bones and the brain. Metastases in soft tissues such as fat, muscle, tendons and connective tissue are rare, occurring only in 1.8% of cancer patients [8]. Due to this low incidence, they are rarely reported in literature [9]. El Abiad et al. [10] reported that adenocarcinoma is the most common histologic subtype, followed by squamous cell carcinoma. Plaza et al. [11] found that the most common type of metastases to soft tissue were carcinomas (70%), followed by metastases of malignant melanoma and sarcoma. Carcinoma that metastasize to soft tissue most often originates from lung cancer, followed by kidney and colon cancers [12].

The first complaints of soft tissue metastases are often nonspecific pain complaints, leading to delayed diagnosis. As a result, soft tissue metastases are often large by the time they cause symptoms like pain, discomfort and loss of function. In addition to site-specific complaints, patients also experience systemic symptoms including fatigue, shortness of breath and loss of appetite.

Treatment of soft tissue metastases from any type of malignant tumors is multidisciplinary and consists of chemotherapy in case of widespread metastases and local intervention in patients with symptomatic lesions and good performance status [10]. Local treatment with palliative radiotherapy can reduce pain and improve symptoms. In patients with bone metastases a lot is known about response rates, duration of pain relief and improvements in quality of life (QOL) after radiotherapy [13]. Palliation of painful bone metastases improves QOL problems directly related to the pain and as a result physical problems [14]. In fact, 60–80% of patients experience decreased pain relief and 30–50% experience disappeared pain within 3–4 weeks of starting radiotherapy. Use of radiotherapy for metastases in soft tissue lesions has been rarely reported [15]. Bae et al. [16] reported that palliative radiotherapy achieved relief from symptoms in 80% of patients with a symptomatic pelvic mass from metastatic colorectal cancer. When initiating radiotherapy key considerations include patient wishes, prognosis, QOL and side effects. Choosing the right radiotherapy treatment is difficult. There is evidence to suggest that radiation oncologists are poor prognosticators, invariably overestimating expected survival [17]. As a result of the generally poor prognosis of these patients, identifying relevant prognostic factors is essential for adequate treatment. However, the prognostic factors for patients with soft tissue metastases have not been clarified [18]. The aim of this study was to determine the overall result of treatment with palliative radiotherapy for patients with soft tissue metastases and to identify prognostic factors for overall survival.

## Methods and materials

From September 2015 to April 2023, 1800 patients were included in the database of the palliative radiotherapy clinic of the Erasmus MC Cancer Institute's Department of Radiation Oncology in Rotterdam, the Netherlands. They provided informed consent to participate in this prospective study. From this group: 120 patients (6,7%) with soft tissue metastases were included in this study. This study was approved by the institutional medical ethics committee (METC) of the Erasmus MC with approval number MEC-17–230.

Patients with a soft tissue metastases invading the bone were also included if the largest part of the tumor was outside the bone. Mesothelioma or lymphoma were excluded. During the consultation with the radiation-oncologist patient demographics, medicine use and detailed information about disease status were collected. External beam radiotherapy was given in different fractionation schedules. Eighty eight patients were treated with 20 Gy in 5 fractions, 21 with 30 Gy in 10 fractions and 11 patients with 8 Gy in 1 fraction or 16 Gy in 2 fractions. 2D, 3D or Vmat planning was used applied with a linear accelerator. The planning target volume (PTV) consisted of the clinical target volume (CTV) + 8 mm. CTV = gross target volume (GTV) + 5 mm. Dose was calculated in Monaco planning system, version 5.11.02 to 6.00.01. Complaints such as pain, swelling, functional disability and other complaints were noted in the electronic patient file by the radiation oncologist during the first patient visit. Four or eight weeks after the last fraction, the patient themself was asked about the outcome of the treatment. During a telephone consultation by the radiation oncologist at 4 weeks after last fraction or the radiation therapy technologist (RTT) at 8 weeks after last fraction, the patient was asked the question: 'did you benefit from the treatment?' The only two possible answers to this question were: 'yes' or 'no', and this was written down in the electronic medical file. If the patient answered positively 4 or 8 weeks after last fraction, then they were classified as responders and non-responders otherwise. The change in pain, swelling, functional disability and other complaints were noted in the electronic patient file by the radiation oncologist or the RTT. Overall survival was calculated from date of the first treatment fraction to date of death or date of last follow-up as recorded in the electronic patient file. The survival data were retrieved from the medical record, population register or by contacting the general practitioner. Survival and local control were estimated using the Kaplan-Meier method and the log-rank test was used to test for differences.

Based on literature, 21 factors were collected with a possible impact on patients survival and subdivisions were made to simplify interpretation (Table 1).The following 12 prognostic factors were considered of interest for further analysis: 1) Karnofsky score, 2) number of organs metastasized, 3) number of metastases 4) morphine use, 5) extra care 6) time from diagnosis to treatment, 7) time from first metastasis to treatment 8) place of stay during radiotherapy, 9) treatment options after radiotherapy, 10) synchronous metastases, 11) curative treatment in history, 12) PTV-volume.

**Table 1:** Patient characteristics.

**Patient characteristics (n = 120)**	**Nr. of patients (%)**
**Age**	
0–70	76 (63)
>70	44 (37)
**Sex**	
Female	43 (36)
Male	77 (64)
**Primary tumor**	
Lung	43 (36)
Colorectal	17 (14)
Other	60 (50)
**KPS**	
<80	59 (49)
100–80	61 (51)
**Liver metastases**	
Yes	22 (18)
No	97 (81)
Unknown	1 (1)
**Lung metastases**	
Yes	67 (56)
No	53 (44)
**Number of organs**	
1–2	61 (51)
>2	59 (49)
**Number of metastases**	
1–3	18 (15)
4–10	47 (39)
>10	55 (46)
**Morphine**	
Yes	71 (59)
No	49 (41)
**Extra care**	
Yes	19 (16)
No	86 (72)
Unknown	15 (12)
**Date of first diagnosis**	
0–3 years	88 (73)
>3 years	32 (27)
**Date of first metastasis**	
0–3 years	103 (86)
>3 years	17 (14)
**Place of stay**	
Home	107 (89)
Hospital	10 (8)
Unknown	3 (3)
**Systematic treatment after RT**	
Yes	34 (28)
No	86 (72)
**Location**	
Head/neck	22 (18)
Thorax	40 (34)
Abdomen	53 (44)
Extremities	5 (4)
**Systemic therapy before RT**	
Yes	40 (33)
No	77 (64)
Unknown	3 (3)
**Synchronous metastases**	
Yes	60 (50)
No	60 (50)
**Curative treatment in history**	
Yes	69 (58)
No	51 (42)
**Risk of fracture**	16 (13)
Yes	101 (84)
No	3 (3)
Unknown	
**Brain metastases**	11 (9)
Yes	107 (89)
No	2 (2)
Unknown	
**PTV-volume (cc)**	25 (21)
0–249	
250–750	59 (49)
>750	36 (30)

Cox proportional hazard models were used to estimate hazard ratios (HR), with 95% confidence interval (CI) and p-values. Covariates with a p-value of ≤ 0.20 in a univariable analysis were entered into a multivariable model. A two sided significance level of 0.05 was used for all other tests. Substantial collinearity was observed between presence of synchronous metastases, time interval from diagnosis to start radiotherapy, and time interval from first metastasis to start radiotherapy. Akaike’s information criterion (AIC) was used to select which of these three parameters should best be entered into the model, where the time intervals were log transformed (with base 2) to make them more normally distributed. A model with time since diagnosis and synchronous metastases fitted best according to the criterion. Other covariates were kept in the final multivariable model if they stayed significant at the 0.20 level in a backward selection procedure. All analyses were performed using the IBM SPSS Statistics version 28.0 software package (SPSS Inc., Chicago, IL, USA) and R version 4.4.2 (R Foundation for Statistical Computing, Vienna, Austria).

### Ethical statement

This study was approved by the institutional medical ethics committee (METC) of the Erasmus MC with approval number MEC-17–230.

## Results

Between September 2015 and April 2023, 120 patients were enrolled within this study. The most common primary tumor was lung cancer (36%), followed by colorectal cancer (14%). The remainder contained patients with primary tumors as bladder, breast and sarcoma. Twenty-two patients had metastases in the head and neck region, 40 in the thorax, 53 in the abdomen and 5 in the extremities. Seventy one patients used morphine before the start of the treatment and 34 patients had other systemic treatment options after radiotherapy. Most patients (91%) were treated with multiple fractionation schedules (5–10 fractions). All patient characteristics are summarized in Table 1. In total, the 120 patients had 152 complaints of advanced cancer that were the reason for treatment: 104 pain, 20 uncomfortable tumor mass, 19 functional disability and 9 other complaints. The result of the treatment on the 152 complaints was a decrease or disappearance in 93 of 152 complaints (61%). In 41 of 152 complaints (27%) there was no effect of the treatment, and the effect on 18 complaints (12%) was unknown.

Pain was the most common complaint and was reported in 104 patients (87%). Pain disappeared or decreased in 63 patients (61%). 20 patients (17%) were treated for an uncomfortable tumor mass. None of the patients did report a complete disappearance but the tumor mass decreased in 60% of patients which resulted in improvement of function and/or reduction of pain. 19 patients (16%) were treated with radiotherapy for functional disability. Functional disability decreased or disappeared in 58% of complaints. These patients reported improvement in strength and mobility. Nine patients (8%) had other complaints, such as melena, edema, hematuria, blood loss, cough or a progressive metastasis without symptoms. In 7 patients (78%) the treatment had a positive effect on complaints and in 3 patients the complaints disappeared completely. Table 2 shows the outcomes reported by the patients.

**Table 2:** Outcome of complaints at 8 weeks after last fraction.

**Reason for treatment**	**N**	**Disappeared**	**Decreased**	**No/negative effect**	**Total effect of RT(%)**
**Pain**	104	13	50	32	63 (61)
**Swelling**	20	0	12	2	12 (60)
**Functional disability**	19	1	10	5	11 (58)
**Other**	9	3	4	2	7 (78)

Median overall survival of the whole group was 4.3 months (95% CI 2.4–9.3). The six-month survival was 37% (95CI 29% − 46%) and the one year survival was 12% (95CI 7% − 19%). At moment of analysis 115 patients had died. Median survival is listed in Table 3 per category. Median follow up of patients alive at the time of analysis was 27 months.

**Table 3:** Univariable analysis of overall survival.

		**Kaplan Meier**	**Cox Regression (univariable)**	
		**Median OS**		
		**Months**	**HR**	***p-value***
**1**	**Age**			0.8
	0–70	4.2		
	>70	3.4	1	0.8
**2**	**Gender**			
	Female	4.2		
	Male	4.2	0.77	0.2
**3**	**Primary tumor**			0.7
	Lung	3.8		
	Colorectal	5.9	1.26	0.4
	Other	4.2	1.27	0.4
**4**	**KPS**			
	<80	3.3		
	100–80	5.5	0.62	0.012
**5**	**Liver metastases**			
	Yes	3.8		
	No	4.2	0.81	0.4
**6**	**Lung metastases**			
	Yes	3.8		
	No	5.5	0.89	0.5
**7**	**Number of organs**			
	1–2	5.3		
	>2	3.8	1.45	0.056
**8**	**Number of metastases**			0.005
	1–3	4.3		
	4–10	5.5	1.49	0.2
	>10	3.4	2.38	0.004
**9**	**Morphine**			
	No	3.8		
	Yes	5.4	1.49	0.041
**10**	**Extra Care**			
	Yes	2.5		
	No	4.5	0.61	0.059
**11**	**Time from diagnosis to RT date**			
	0–3 years	3.7		
	˃3 years	8.2	0.93	0.002
**12**	**Time from metastasis to RT date**			
	0–3 years	3.8		
	˃3 years	6.6	0.9	0.013
**13**	**Place of stay**			
	Home	4.2		
	Hospital	2.5	1.66	0.13
**14**	**Treatment options after RT**			
	Yes	6.6		
	No	3.6	1.6	0.026
**15**	**Location**			0.4
	Head/neck	4.6		
	Thorax	4.6	0.93	0.8
	Abdomen	3.7	1.31	0.3
	Extremities	3.8	1.1	0.8
**16**	**Systemic therapy before RT**			
	Yes	5.5		
	No	3.8	0.98	>0.9
**17**	**Synchronous metastases**			
	Yes	3.7		
	No	5.5	0.53	0.001
**18**	**Curative treatment in history**			
	Yes	4.6		
	No	3.8	1.55	0.023
**19**	**Risk of fracture**			
	Yes	4.3		
	No	4.2	1.07	0.8
**20**	**Brain metastases**			
	Yes	2		
	No	4.2	0.75	0.4
**21**	**PTV volume (CC)**			0.033
	0–249	4.2		
	250–750	5.5	0.68	0.11
	>750	3.8	1.18	0.5

**Fig. 1:**
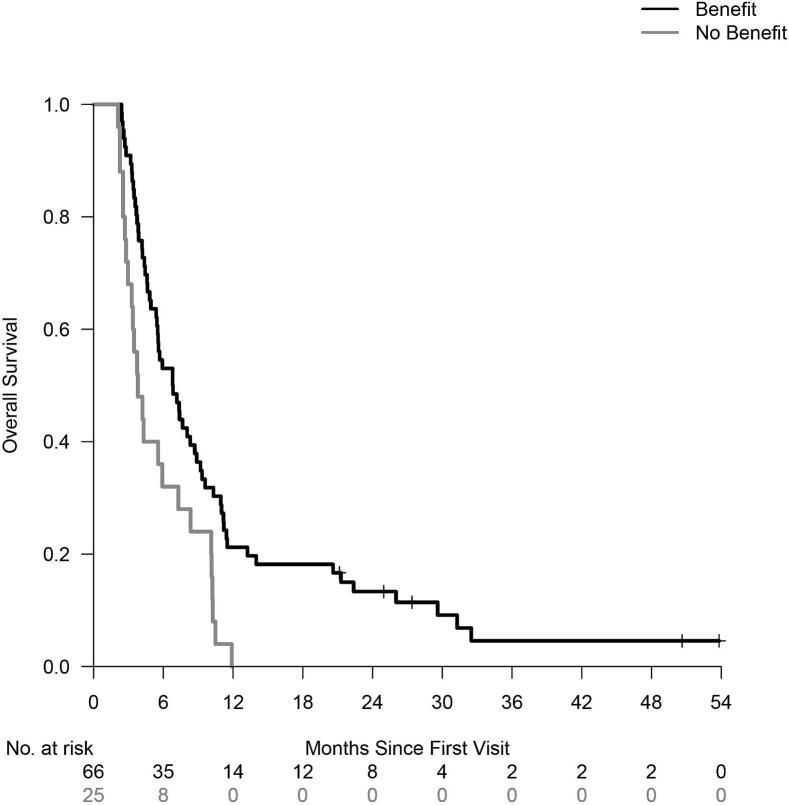
Overall survival in patients with or without benefit at any time 4 and 8 weeks after radiotherapy (subgroup for patients alive at 2 months after start of treatment).

Two months after the treatment, 94 patients were still alive (78%, 95% CI 71%–86%). There were 66 responders, 25 non-responders and the answer was unknown in 3 patients. Responders had a median overall survival of 6.6 months compared to 3.8 months for non-responders (p = 0.005, Fig. 1). Of the 26 patients who died before 2 months after the treatment, there were 8 responders and 9 non-responders. The response of the treatment was unknown in 9 patients. In 34 non-responders who answered negatively to the question whether there was benefit, 31 patients were treated for pain. In 16 of these 31 non-responders the treatment had no effect on the pain. The remaining 15 non-responders reported variable pain (5 patients), insufficient decrease (5 patients), no properly pain reduction due to other pain not in the treated area (3 patients). Two patients noticed improvement in strength or disability of function, but still scored the result of the treatment insufficient because the pain remained unchanged.

**Fig. 2:**
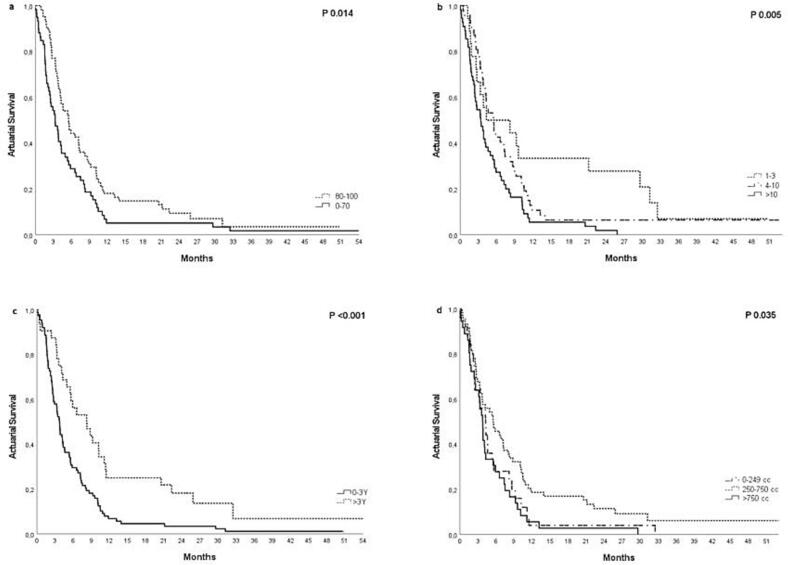
Overall survival according to treatment options after radiotherapy (a), number of metastases(b), date of first diagnosis(c) and PTV-volume(d).

In univariable analysis (Cox regression) twelve parameters of the 21 were significant for overall survival (Table 3). Collinearity was observed between the three parameters time from diagnosis to treatment, time from metastases to treatment and synchronous metastases. Time from diagnosis to treatment and synchronous metastases were selected to be included in the multivariable analysis based in the AIC. A multivariable analysis was performed, 5 factors remained significant: 1) PTV-volume, 2) time from diagnosis to treatment, 3) total number of metastases, 4) systemic treatment options after radiotherapy, (5) number of organs metastasized (Table 4). Fig. 2 shows the Kaplan Meier curves of the total group of 120 patients of four significant prognostic factors for survival: treatment options after radiotherapy, number of metastases, time from diagnosis to treatment and PTV-volume.

**Table 4:** Multivariable analysis of overall survival.

	**HR**^a^	**95% CI**^a^	**p-value**
**PTV volume (CC)**			<0.001
1–249			
250–750	0.54	0.33, 0.90	0.019
>750	1.59	0.90, 2.80	0.11
**Time from diagnosis to RT date**	0.82	0.74, 0.90	<0.001
**Number of metastases**			0.002
1–3			
4–10	1.45	0.75, 2.81	0.27
>10	2.76	1.40, 5.45	0.003
**Treatment options after RT**			0.008
Yes			
No	1.85	1.16, 2.97	0.01
**Number of organs**			0.18
1–2			
>2	1.36	0.87, 2.14	0.18
**Supportive care**			0.2
Yes			
No	0.69	0.40, 1.19	0.18

a HR = Hazard Ratio, CI = Confidence Interval.

## Discussion

Sufficient literature is available on prognostic factors and the effect of radiotherapy to reduce symptoms in patients with bone and brain metastases [14], [19], [20], [21]. In contrast, available literature on the outcome of radiotherapy in patients with soft tissue metastases is limited. Case series found in the literature mostly involved soft tissue metastases in skeletal muscle and originated from primary lung tumors [11], [22], [23]. This is similar to our study in which the most common primary tumor was also lung carcinoma. We included 120 patients (6.7%) out of our data collection of 1800 patients over a period of almost 8 years. Studies reporting palliative radiotherapy on soft tissue metastases are often limited due to the small number of included patients or extend over a very long period of time [9], [10], [11], [15], [19], [23], [24], [25]. Vargas et al. [15] examined whether a single dose is sufficient palliation in only 4 patients with soft tissue metastases. Tuohetti et al.[23] and Herring et al.[24] based their studies on only 12 and 15 patients respectively. Tsuchie et al. [25] reported on 16 patients over a period of 24 years. Plaza et al [11] reviewed 118 patients with a diagnosis of metastases in soft tissue from a total of 7237 in a period of 29 years between 1971 and 2000.

The most commonly reported symptom for treatment was pain (87%). In addition, patients received treatment for other complaints such as bleeding, edema, feeling of a tender, uncomfortable spot, reduced mobility and functional limitations.

The total effect of treatment in our study scored by the patient (61% decrease or disappearance of complaints) showed responders had a significantly better survival compared to non-responders. Nine patients with an unknown outcome died within 2 months and did not have enough survival time to report at 8 weeks. They had a median survival of 3 weeks and may have been responders after 4 weeks. Because this remained unknown, we considered them as non-responders. We found no other comparable study in the literature where patient reported outcomes were used.

Patients with a soft tissue metastasis often have a swelling without symptoms for a long time which can finally present as painful masses [9], [22], [23], [24]. In more than half (59%) of patients in our study, the time between the date of palliative radiotherapy and the development of first metastases was > 6 months, and in 14% of patients it was even > 3 years. Multiple fractions are more often given in patients with soft tissue extension to achieve local control of the metastasis in addition to pain relief [20], [26]. In our study, 91% of patients were treated with multiple fractionation schedules (5–10 fractions). Saito et al. [27] reported that there might be a dose–response relationship in radiotherapy for painful non-bone lesions. Higher total radiation dose seemed to be associated with a higher rate of pain response in patients with non-bone painful lesions. However, univariate and multivariate Cox regression analyses showed that total radiation dose was not a significant predictor of pain response [27]. Due to the small group of patients in our study treated with a single fraction (9%), no evidence was found for a dose–response relationship.

In our study, 22% of patients died within two months and median overall survival was 4.2 months. A comparable survival duration from diagnosis of soft tissue metastases to death was 5.4 months (range 1–19 months) in the study of Damron et al. [9] Patients were treated with radiotherapy, chemotherapy, excision or a combination of these. The most common presenting symptom was a painful mass, similar to our study. Bae et al. [16] reported a median survival of 6 months in his study with patients who were treated to a symptomatic pelvis mass with palliative radiotherapy. In a study focusing on soft tissue metastases from esophageal cancer, a higher median overall survival of 8.9 months was reported. In patients who underwent local radiotherapy, 4 of 26 had a median survival of 13.3 months [10]. Given this small number of patients we can't compare this with our study.

We found that patients with a PTV volume between 250 and 750 cc had better median overall survival (5.5 months) compared to the groups with a PTV below 250 cc (4.3 months) and above 750 cc (3.8 months) and this difference was significant. Nieder et al. [28] showed in patients with lung cancer who received radiation in the thorax that the volume of the target was not significantly associated with survival. This suggests that large size should not preclude palliative radiotherapy as long as normal tissue dose constraints can be achieved.

A limitation of this study is that the effect of the treatment could be influenced by symptoms other than those for which the patients were treated. Patients with metastases often experience fatigue, nausea, cough, pleural effusion, edema, weight loss or malaise. Some patients did notice that the treatment increased their strength or improved their function, but because the pain did not decrease, they judged the treatment as insufficient, even though radiation did have a positive effect.

Another limitation is that the reduction of complaints were not assessed using a validated grading system, but was instead reported by the patients themselves. Since symptom reduction is the primary outcome measure for the patient, self-reporting can be susceptible to subjective biases, such as selective recall or an overestimation of improvement. To increase reliability, comparability and measurability, it is recommended to use validated measurement instruments in future research.

## Conclusion

Palliative radiotherapy for patients with soft tissue metastases provided benefit from complaints in 61% of patients and these had a significantly better survival than patients who reported no benefit. Although 5 prognostic factors for survival were identified, further research is needed to identify patients who benefit from palliative radiotherapy.

## CRediT authorship contribution statement

**Lijanne Otto-Vollaard:** Conceptualization, Data curation, Formal analysis, Investigation, Methodology, Project administration, Validation, Visualization, Writing – original draft, Writing – review & editing. **Marcella Cramer:** Data curation, Investigation, Writing – review & editing. **Erik van Werkhoven:** Formal analysis, Methodology, Validation, Writing – review & editing. **Ingrid Lokken:** Data curation, Investigation, Writing – review & editing. **Ilse M.N. de Pree:** Conceptualization, Investigation, Project administration, Writing – review & editing. **Joost J. Nuyttens:** Conceptualization, Data curation, Formal analysis, Investigation, Methodology, Project administration, Supervision, Validation, Visualization, Writing – original draft, Writing – review & editing.

## Declaration of competing interest

The authors declare the following financial interests/personal relationships which may be considered as potential competing interests: The department of radiotherapy of the Erasmus MC has a research contract with Electa, Varian and Accuray.

## Data Availability

Research data are stored in an institutional repository and can be shared upon request to the corresponding author.
